# An Efficient Compressive Sensing Event-Detection Scheme for Internet of Things System Based on Sparse-Graph Codes

**DOI:** 10.3390/s23104620

**Published:** 2023-05-10

**Authors:** Jun Cai, Xin Xu, Hongpeng Zhu, Jian Cheng

**Affiliations:** College of Communications Engineering, Army Engineering University of PLA, Nanjing 210007, China

**Keywords:** event detection, integer-valued signal, IoT, sparse graph codes, compressive sensing

## Abstract

This work studied the event-detection problem in an Internet of Things (IoT) system, where a group of sensor nodes are placed in the region of interest to capture sparse active event sources. Using compressive sensing (CS), the event-detection problem is modeled as recovering the high-dimensional integer-valued sparse signal from incomplete linear measurements. We show that the sensing process in IoT system produces an equivalent integer CS using sparse graph codes at the sink node, for which one can devise a simple deterministic construction of a sparse measurement matrix and an efficient integer-valued signal recovery algorithm. We validated the determined measurement matrix, uniquely determined the signal coefficients, and performed an asymptotic analysis to examine the performance of the proposed approach, namely event detection with integer sum peeling (ISP), with the density evolution method. Simulation results show that the proposed ISP approach achieves a significantly higher performance compared to existing literature at various simulation scenario and match that of the theoretical results.

## 1. Introduction

Recently, compressive sensing (CS)  [1,2] theory has been widely used in the Internet of Things (IoT) system [3,4,5,6] and has shown significant performance improvements in network life, energy efficiency, and overall system throughput. CS theory is profitable to IoT system since it usually has limited perception, limited memory, and a small communication bandwidth and capacity [7,8,9].

In this paper, we investigate how to employ CS in wireless sensor networks, which involve a large number of sensor nodes. A typical scenario is an event-detection problem in the wireless sensor networks where a group of monitoring sensors try to capture few active event sources from many event resources. Specifically, we focus on two issues of wireless sensor networks. First, compared with the total number of sensors in the network, the number of active sensors is very limited. In addition, the number of events is much smaller than the number of all sources. Second, different events may occur at the same time and cause interference to detect them separately. As a result, the received signals are all superimposed, and an effective algorithm is needed to separate the signal $x\in R^{n}$.

Generally, consider the estimation of an unknown real-valued signal vector $x\in R^{n}$ from a vector of linear measurement $y\in C^{m}$ (m<n), i.e.,

(1) y=Hx,

where $H\in C^{m\times n}$ is often referred to as a measurement matrix, the number of nonzero elements of *x* is small, k≪n, and *k* is known as the sparsity of *x*.

More specifically, we show that the sensing process in an IoT system produces an equivalent integer CS using sparse graph codes at the sink node. For this, one can devise a simple deterministic construction of the sparse measurement matrix and an efficient integer-valued signal recovery algorithm. Furthermore, many practical IoT systems can be modeled as integer CS based on sparse measurement matrices.

Similarly to traditional CS, the two main concerns in the integer CS of IoT systems are: (1) designing the measurement matrix *H* and (2) recovering x from the measurements y by exploiting the sparsity constraint and integer property of x.

In this paper, we focus on deterministic as opposed to random measurement matrices for integer CS of IoT systems. The deterministic matrices are useful since in practice, the matrix *H* in Equation (1) has to be a deterministic matrix. Furthermore, by designing the proper matrix, we can improve certain features on CS of IoT systems such as recovery computational complexity and compression ratio.

Specifically, we mainly study the construction of the sparse measurement matrix and the reconstruction algorithm with integer CS based on sparse-graph codes. On the one hand, the sparse measurement matrix and reconstruction algorithm based on sparse-graph codes simplifies the construction process and lowered the measurement dimension, which is convenient for practical application.

The construction of measurement matrix must be guaranteed that each measurement can uniquely reconstruct the original signal. For example, suppose $h_{1}+h_{2}=h_{3}$, where $h_{1},\;h_{2},\;h_{3}$ is the linear projection coefficient. If the binary signal x is measured, the measurement is denoted as $h_{1}x_{1}+h_{2}x_{2}+h_{3}x_{3}=h_{3}$. However, two possible solutions are obtained by measurement: $x_{1}=x_{2}=0$, $x_{3}=1$ and $x_{1}=x_{2}=1$, $x_{3}=0$. Obviously, the matrix design does not meet the uniqueness requirement. In this paper, we mainly focus on the construction of deterministic sparse matrices and their reconstruction algorithms for integer CS of IoT systems. Our idea comes from the concept of algebraic numbers and their minimum polynomials in number theory, which ensures the uniqueness of integer signal reconstruction. The main contributions of this paper are as follows:

(1) Using the sparse-graph codes in channel coding and integer CS of IoT systems, we design a new class of deterministic sparse measurement matrices which are sufficient to guarantee the unique recovery for integer CS of IoT systems.
(2) By exploiting the integer-valued structure of the event-detection signal and sparse measurement matrix, an efficient event-detection signal recovery algorithm is proposed. Furthermore, the asymptotic analysis is also conducted on the performance of the recovery algorithm with the density evolution method.
(3) In order to deal with the noise, the above-mentioned measurement matrix design is extended to the noisy situation, and an effective reconstruction criterion under the noise condition is proposed.

Throughout this paper, we use the following notations. For a vector x, $x_{i}$ denotes the the *i*-th entry of x. For a matrix *H*, we use $h_{i}$ to denote the *i*-th row of matrix *H*, the transpose of matrix *H* is denoted by $H^{\prime }$. For any support set Γ, the cardinality of Γ is denoted as $\left|\Gamma \right|$, $x_{\left\{\Gamma \right\}}$ denotes a vector of length $\left|\Gamma \right|$ taken from x with support set Γ.

## 2. Related Work

Several approaches have used CS theory for event detection problem. For example, the multivariated Bayesian compressive sensing (BCS) approach has been adopted in [10] to settle the event detection problem in the IoT system; however, the multivariated BCS approach is known to be sensitive to noise, resulting in poor performance in low signal-to-noise ratios (SNRs). To settle this problem, Eltabie et al., in [11], proposed the use of assisted compression to model the dense IoT problem. However, massive sensor nodes must be employed in the sensing field to guarantee its performance. Generally, the event detection problem in IoT system can be modeled as a integer compressive sensing problem.

In traditional CS, the sparse signals are defined over the field of real numbers. However, in practice, the alphabet of the sparse signal can be modeled as a finite set of integers. For example, blind estimation in digital communications [12], binary sparse signals [13,14], and other counting data sets, including arrivals at a queue/server, are inherently integer-valued. In such cases, it is called integer CS [7,15,16], where knowledge of the sparsity and the integer property can prove to be useful.

Most of the measurement matrices in the event-detection problem of the IoT system are based on randomization, such as the i.i.d Gaussian random matrix [7,17]. In general, the exact solution to the second concern is shown to have a combinatorial nature and the computational time becomes exponential [17]. However, if both the sparsity and the integer-valued features are exploited, some efficient algorithms, such as sphere decoding and CVX in convex programming, can be performed. In this case, the exact exhaustive search with the same solution for almost all the possible inputs would not be necessary [13,17].

Most of the works on event-detection algorithms of the IoT system are based on the random matrix [13,14,18]. In [13,14], the authors focused on the binary sparse signal. The problem of detecting the entries of binary signal was formulated as a Bayesian framework in [13]. M. Shirvanimoghaddam et al. proposed a binary signal-recovery approach based on analog fountain codes. In [7], the authors provided a link between superimposed codes (SC) and integer CS, where the new codes, termed as weighted superimposed codes (WSC), were proposed.The number of required signal measurements in WSC is of O(klogn/logk), which is less than the standard O(klog(n/k)). In [17], to reduce the computational complexity of the recovery, the authors solved the problem by minimizing the sum of weighted absolute values. However, the probability distribution defined on an alphabet should be known. In [19,20,21,22], the authors considered compressed sensing over a finite alphabet, where the elements of the measurement matrix are also in a finite alphabet. In this paper, we revisit the event-detection problem in an IoT system from the coding theory perspective and solve the problem as a decoding problem.

## 3. System Model and Problem Formulation

### 3.1. System Model

**Figure 1:**
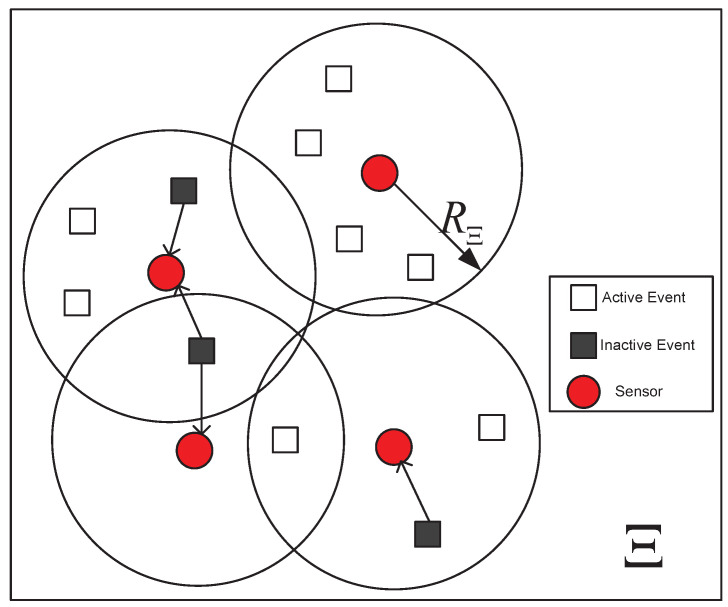
*m* active sensors are capturing *k* out of *n* active events, where $R_{\Xi }$ is the monitoring range and the size of arrow indicates different $R_{\Xi }$.

The *n* event sources in the IoT system, denoted as $E_{1},\;E_{2},\;\cdots ,\;E_{n}$, are randomly distributed in the detection area Ξ, as shown in Figure 1. Each event source $E_{i}$ randomly generates an event $x_{i}$ which can be detected by the sensor nodes in the area. Since only the active state of the event source is detected, the event $x_{i}$ is defined as a binary signal, indicating that the event $x_{i}=1$ is an active event, and the event $x_{i}=0$ is an inactive event. Note that the number of simultaneous active events is *k* (k≪n), so the vector x is a *n*-dimensional *k*-sparse binary vector.

Assuming that there are *m* sensors used to monitor the event source in the area, its monitoring range is $R_{\Xi }$, then each sensor can monitor the event source of the distance sensor at most $R_{\Xi }$. Denote $h_{i,j}$ as the channel of the *i*-th event source and the *j*-th sensor, then the signal $y_{j}$ received by the *j*-th sensor is:

(2) $$y_{j}=\sum_{i\in \Xi_{j}}h_{i,j}x_{i}+n_{j},$$

where $\Xi_{j}$ is the event source within the monitoring range of the *j*-th sensor, and $n_{j}$ is noise. Its vector form is:

(3) y=Hx+n.

Generally, sensors are randomly distributed in the monitoring area; event sources that are not within the monitoring range cannot be monitored. In other words, when the total monitoring area Ξ is large, most elements $h_{i,j}$ are zero, thus, the matrix H is sparse. Therefore, when the number of sensor nodes is less than the number of event sources (m<n), the monitoring model of active events in the wireless sensor network can be regarded as an integer compressed sensing model based on a sparse measurement matrix. In addition, the detection of the positioning signal in GPS system can also be regarded as a sparse matrix and an integer compressed sensing model.

### 3.2. Problem Formulation

Generally, the event detection problem in IoT systems can be modeled as a integer CS problem. Given k,n,q∈N with k≪n, and a finite alphabet $X=\{\chi_{1},\;\chi_{2}\;\cdots \;\chi_{q}\}\subset Z$ with 0∈X and $\left|X\right|=q$, we consider the following ensemble:

$$S=\{x=\left(x_{1},\;x_{2},\;\cdots ,\;x_{n}\right)\in X^{n}:{∥x∥}_{0}=k\}$$

This ensemble represents the space of *n*-dimensional signals that are *k*-sparse with entries coming from S.

In [17], the authors discussed the uniqueness of the solution of the discrete signal recovery. If the pair (X,H) is chosen to satisfy:

(4) $$\ker H\cap {\tilde{X}}^{n}=\left\{0\right\},$$

where $\ker H≜\{x\in C^{n}:Hx=0\}$ and $\tilde{X}≜\left\{r_{i}-r_{j}:i,j=1,2,\cdots q\right\}$, then the linear equation associated with measurements in (1) have a unique solution.

As long as the uniqueness assumption holds, the exact solution can be obtained in a finite number of steps by enumerating all possible $X^{n}$ in ensemble S. By denoting $X^{n}=\left\{x_{1},\;x_{2},\;\cdots ,\;x_{\mu }\right\}$, we can uniquely determine the values of x in a finite time by comparing the measurement vector y and $Hx_{i}$ (1⩽i⩽μ). However, the computational complexity of this enumerating computation is exponential. For example, when q=3 and n=1000, the number of comparisons is $\mu =3^{1000}$ in the worst case.

To reduce the computational complexity, we borrow the insights of sparse graph codes in channel coding and CS [23,24]. Our basic design is that if each row of *H*, say $h_{l}$, is sparse (most of the elements in $h_{l}$ are zero), then each measurement $h_{l}x$ may only involve few nonzero elements of x since most of elements of x are also zero; thus, the size of the problem is reduced significantly, and the few nonzero elements of x can be recovered by the efficient enumerating method in an iterative manner.

## 4. Event Detection Using Sparse Graph Codes

This section first briefly presents a bipartite graph. Then, we explain that the event-detection problem in the IoT system is a special case of sparse graph decoding, so it can be solved with fewer measurements, by the standard peeling decoding algorithm, compared with the existing methods. We further design the measurement matrix and obtain the lower bound of the minimum number of sensor nodes required to successfully capture all active events.

### 4.1. Preliminaries

Any sparse matrix can be efficiently represented by a bipartite graph. Let G=(V∪C,∂) denote a bipartite graph with variable nodes set V, check nodes set C, and the edge set *∂*; every edge in *∂* connects a node $v_{i}$ in V to a node $c_{i}$ in C. Additionally, let $\tilde{H}$ denote the bi-adjacency matrix of bipartite graph *G*:

$${\tilde{H}}_{i,j}=\left\{\begin{array}{c} 1,\mathrm{if}\{v_{i},c_{j}\}\in \partial \\ 0,\mathrm{if}\{v_{i},c_{j}\}\notin \partial \end{array}\right.$$

In a bipartite graph, a node in C(V) has degree *i* when it connects to *i* nodes in V(C). Let $\lambda_{i}$ denote the fraction of nodes with degree *i* in V, $\rho_{j}$ denote the fraction of nodes with degree *j* in C, and let

$$\lambda \left(x\right)=\sum_{i}\lambda_{i}x^{i-1},\rho \left(x\right)=\sum_{j}\rho_{j}x^{j-1}$$

represent the degree distributions.

When the measurement matrix is sparse, the event detection problem in the IoT system also can be represented by a bipartite graph, where the sets of signal coefficients and measurements are mapped to the variable nodes set V and check nodes set C, respectively. In this paper, we denote the set of variable nodes that incident to check node *l* by $N\left(l\right)=\left\{i:h_{l,i}\neq 0\right\}$.

Since the event source generates integer-value signals, the event vector can be regarded as integer-value symbols during sparse graph coding. During sensing, the vector is multiplied by the channel matrix *H* and sent to the sink node for further processing. Since we assume that each sensor node only receives signals from event sources in the sensor coverage area, the nonzero entries in each row of *H* are very small. This is very similar to the sparse graph coding process, where the generator matrix is now the channel matrix and the weight coefficient is the channel gain. More specifically, the degree of each coding symbol (measurement or sensor) is represented by the sensor degree, which is now the number of event sources. In addition, the number of nonzero elements in the corresponding row of the generator (or measurement) matrix is determined by these sources. By treating each event source (information symbol) and sensor reading (coding symbol) as variables and check nodes, respectively, the measurement process in the IoT system can be represented by a weighted bipartite graph. The construction of measurement matrix is given as follows.

### 4.2. Measurement Matrix Construction

We construct the measurement matrix using the row tensor product given in [25].

**Definition** **1** **(row tensor product).** *Let A be a $t_{1}\times n$ matrix and B a $t_{2}\times n$ matrix, with rows $\left\{A_{i}:1\leq i\leq t_{1}\right\}$ and $\left\{B_{j}:1\leq j\leq t_{2}\right\}$, respectively. The row tensor product $S=A{⊗}_{r}B$ of A and B is a $t_{1}t_{2}\times n$ matrix whose rows are $\left\{A_{i}B_{j}:1\leq i\leq t_{1},1\leq j\leq t_{2}\right\}$, where $A_{i}B_{j}$ denotes the component wise product of two vectors of length n.*

The measurement matrix is generated from the sparse bi-adjacency matrix $\tilde{H}$ of the bipartite graph *G*. The main focus of this paper is on the left *d*-regular (all variable nodes have the same degree *d*) bipartite graph, i.e., $\lambda \left(x\right)=x^{d-1}$. The results, however, can be generalized to regular or irregular cases.

**Definition** **2** **(measurement matrix).** *Given a m×n sparse bi-adjacency matrix $\tilde{H}$ of left-regular bipartite graph and a complex vector $f=[1,\cdots ,e^{j2\pi (i-1)/p},\cdots ,e^{j2\pi (n-1)/p}]$, the m×n measurement matrix H is given by*

(5) $$H=\tilde{H}{⊗}_{r}f,$$

*where p≥n is a constant prime.*

To guarantee the exact recovery of all the *k*-sparse integer-valued signals, the measurement matrices should be designed first, i.e., satisfying Equation (4), which is able to uniquely determine the signal coefficients. For the constructed measurement matrix, the following theorem holds:

**Theorem** **1.** *For the measurement matrix H constructed as (5), the condition $\ker H\cap {\tilde{X}}^{n}=\left\{0\right\}$ holds for the pair (X,H).*

To proof this theorem, we need some notations.

**Definition** **3** **(algebraic number minimal polynomial).** *The minimal polynomial of an algebraic number ς is the unique irreducible monic polynomial of smallest degree q(x) with rational coefficients such that q(ς)=0 and whose leading coefficient is 1.*

*For the algebraic number $e^{j2\pi /p}$ (p is a prime), the minimal polynomial is $\Phi_{p}\left(x\right)=\sum_{i=1}^{p}x^{\left(i-1\right)}$.*

**Lemma** **1.** *For $\alpha_{i}\in Z$, the equation $\sum_{i=1}^{p}\alpha_{i}e^{j2\pi (i-1)/p}=0$ holds if and only if for every $\alpha_{i}=\alpha$ (i=1,2,⋯p), α is a constant.*

Next, we prove the Theorem 1 via Lemma 1.

**Proof.**  Take any $\kappa \in \ker H\cap {\tilde{X}}^{n}$. There must exist $x^{\prime },x^{\prime \prime }\in X^{n}$ such that $\kappa =x^{\prime }-x^{\prime \prime }$ as $\kappa \in {\tilde{X}}^{n}$. With the definition of kerH, then we have $H\kappa =H(x^{\prime }-x^{\prime \prime })=0$, i.e., $h_{l}\left(x^{\prime }-x^{\prime \prime }\right)=0$ (1⩽l⩽m). From the construction as in (5), we have

(6) $$\sum_{i\in N(l)}\alpha_{i}e^{j2\pi (i-1)/p}+\sum_{i\notin N(l)}0\cdot e^{j2\pi (i-1)/p}=0,\left(1⩽i⩽p\right),$$

where $\alpha_{i}=x_{i}^{\prime }-x_{i}^{\prime \prime }$ (i∈N(l)) is an integer. From Lemma 1, the Equation (6) holds if and only if $\alpha_{i}=0$, i.e., $x_{i}^{\prime }=x_{i}^{\prime \prime }$ for all i∈N(l). As $\overset{m}{\underset{l=1}{⋃}}N(l)=\left\{1,2,\cdots ,n\right\}$, we have $x^{\prime }=x^{\prime \prime }$. Thus $\kappa =x^{\prime }-x^{\prime \prime }=0$, this implies $\ker H\cap {\tilde{X}}^{n}=\left\{0\right\}$.    □

### 4.3. Integer Sum-Peeling Algorithm for Event Detection

The number of nonzero variable nodes contained in a measurement is called the measurement degree η and denotes $X^{*}=X\backslash 0$. Then, the variable nodes connected to the measurement with η=D (D=0,1) can be verified through the following rules.

**R1.** If ${∥y_{i}∥}^{2}=0$, then all the variable nodes connected to $y_{i}$ are verified as having a zero value.

**R2.** If ${∥y_{i}∥}^{2}={∥\chi_{j}∥}^{2}$ and $l=(∠\chi_{j}y_{i}/2\pi )p$ is an integer, where $\chi_{j}\in X^{*},1⩽j⩽q-1$, then the variable node *l* can be verified with $\chi_{j}$, and all the other variable nodes connected to $y_{i}$ are verified with zero values.

Furthermore, for the nonzero measurement with degree η⩾D (D⩾2), the variable nodes connected to it are verified by the enumeration method.

**R3.** If $y_{i}=\Lambda_{l}f_{\left\{N(i)\right\}}^{{}^{\prime }}$, where $\Lambda_{l}$ is a sparse integer vector of dimension $\left|N(i)\right|$ with greater than 1 but less than *D* different nonzero elements $\chi_{j}$ ($\chi_{j}\in X^{*},1⩽j⩽q-1$), then all the variable nodes connected to $y_{i}$ are verified as $\Lambda_{l}$.

**R1–R3** can be performed since the construction (5) guarantees unique solution. The parameter *D* is called as selective degree. Then, we propose a peeling algorithm called the integer sum-peeling (ISP) algorithm, which is presented in Algorithm 1.


**Algorithm 1** Integer sum-peeling-algorithm**Input:** observation vector y, the measurement matrix *H*, the selective degree *D*, constant prime *p*.
(1) If ${∥y_{i}∥}^{2}=0$ for 1⩽i⩽m, verify: ${\hat{x}}_{\left\{N(i)\right\}}=0$.
(2) If ${∥y_{i}∥}^{2}={∥\chi_{j}∥}^{2}$ and $l=(∠\chi_{j}y_{i}/2\pi )p$ is an integer for 1⩽i⩽m ($\chi_{j}\in X^{*},1⩽j⩽q-1$). Verify: ${\hat{x}}_{l}=\chi_{j}$, ${\hat{x}}_{\left\{N(i)\backslash l\right\}}=0$.
(3) If $0<{∥y_{i}∥}^{2}\neq {∥\chi_{j}∥}^{2}$ for 1⩽i⩽m, $\chi_{j}\in X^{*},1⩽j⩽q-1$ and D>1, set $\tilde{f}={\left[f_{\left\{N(i)\right\}}\right]}^{{}^{\prime }}$, $\bar{\rho }=\left|N(i)\right|$. Construct matrix Λ, its rows consist of all sparse integer vectors of dimension $\bar{\rho }$ with greater than 1 but less than *D* different nonzero elements $\chi_{j}$. Compute the vector: $\zeta =\Lambda \tilde{f}$. Go to step 4).
(4) If $\exists l\;s.t\;\zeta_{l}=y_{i}$, verify: ${\hat{x}}_{\left\{N(i)\right\}}=\Lambda_{l}\;$.
(5) Peel off: remove all the edges connected to verified variable nodes ${\hat{x}}_{\left\{N(i)\right\}}$, $y=y-H\hat{x}$.
(6) Repeat step (1)–(5) until all the variable nodes are verified.**output:** the recovered sparse integer-valued signal $\hat{x}$.



## 5. Performance Analysis of Integer Sum-Peeling Algorithm

This section elaborates on the overall performance of the ISP algorithm using density evolution, which converges to the performance of the tree-like neighborhood $G_{v}^{2\ell }$ as *n* tends to infinity [26,27,28,29]. $G_{v}^{2\ell }$ is defined as the subgraph containing the variable node *v* and all those nodes that are incident to *v* with any path of length less than or equal to 2ℓ.

**Definition** **4.** *A stopping set S is a subset of the set of variable nodes V, by which all neighbors of S are connected to S at least twice.*

### 5.1. Asymptotic Analysis of Integer Sum-Peeling Algorithm

In the following, we provide a definition of the asymptotic analysis, highlighting the technical components.

**Density evolution:** We analyze the performance of our proposed ISP algorithm over a typical graph (i.e., cycle-free) of the ensemble for a fixed number of peeling iterations *i*. We assume that a local neighborhood of every edge in the graph is cycle-free (tree-like), and derive a recursive equation that represents the average density of remaining edges in the pruned graph at iteration.

In this paper, the bipartite graphs are constructed randomly with $\lambda \left(x\right)=x^{d-1}$, i.e., every variable node is connected to *d* check nodes, one uniformly random check node in each of the *d* sets. To analyze the performance of the peeling decoder performed on a tree-like graph, we need to determine another degree distribution $\rho \left(x\right)=\sum_{j}\rho_{j}x^{j-1}$, i.e., $\rho_{j}$. According to the ensemble of left *d*-regular bipartite graph, the degree of a check node is a binomial B(d/m,n) and can be well approximated by a Poisson random variable

(7) $$\rho_{j}\approx \frac{{\left(d/\beta \right)}^{j}e^{-d/\beta }}{j!},$$

where β=m/n is the sampling ratio.

With the tree-like neighborhood, the ISP procedures over different nodes in the first *l* iterations are independent. The ISP algorithm can decode up to *D* nonzero variable nodes, which can be verified by the (l+1)-th iteration if the check node D−1 are connected at most. Let $p_{l}$ be the expected rate of unverified nonzero variable nodes at *l*-th iteration ($p_{0}=k/n$), then, the probability $p_{l+1}$ can be written with regard to the probability $p_{l}$ at the *l*-th iteration in a recursive manner:

(8) $$p_{l+1}=p_{0}{\left(1-\sum_{i=0}^{D-1}\sum_{j}\rho_{j}\left(\begin{array}{c} j-1\\ i \end{array}\right)p_{l}^{i}{\left(1-p_{l}\right)}^{j-1-i}\right)}^{d-1}.$$

More specifically, in this paper we only consider D=1, and 2. When D=1, the term $\sum_{j}\rho_{j}{\left(1-p_{l}\right)}^{j-1}$ in (8) can be approximated by $e^{x}$ of Taylor expansion

(9) $$\rho \left(x\right)=\sum_{j}\rho_{j}x^{j-1}=e^{-\left(1-x\right)\frac{d}{\beta }}/x.$$

Therefore, the density evolution Equation (8) can be obtained as

(10) $$p_{l+1}=p_{0}{\left(1-\frac{e^{-\frac{d}{\beta }p_{l}}}{1-p_{l}}\right)}^{d-1}.$$

Similarly, when D=2, the density evolution equation can be given by

(11) $$p_{l+1}\approx p_{0}{\left(1-\frac{e^{-\frac{d}{\beta }p_{l}}}{1-p_{l}}-p_{l}\frac{e^{-\frac{d}{\beta }p_{l}}}{1-p_{l}}\frac{d}{\beta }\right)}^{d-1}.$$

Table 1 lists the analytical success thresholds of the ISP algorithm for measurement matrix with different *d*, β and *D*. As can be seen, the success thresholds improve with increased *D*. Furthermore, when fixing the sampling ratio β, as shown in Table 1, the overall performance of the ISP algorithm is improved with decreased *d*.

**Figure 2:**
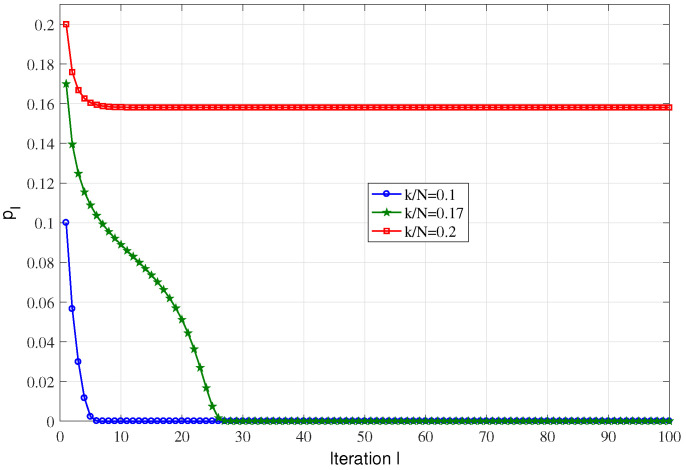
Evolution of $p_{l}$ vs. *l* for the ISP algorithm over (d=3,β=0.2) and D=1.

**Figure 3:**
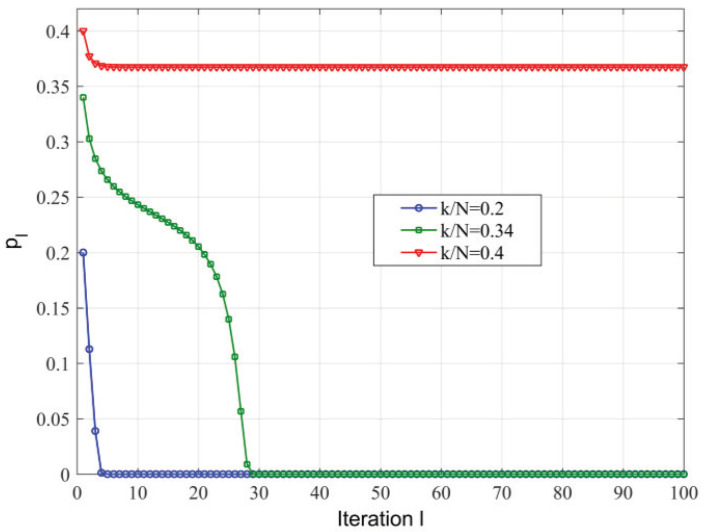
Evolution of $p_{l}$ vs. *l* for the ISP algorithm over (d=3,β=0.2) and D=2.

Our next set of results investigate the degree of agreement between simulation results of ISP algorithm and asymptotic analysis, the plots in Figure 2 and Figure 3 correspond to the evolution of $p_{l}$ with iterations *l* for the ISP algorithm over the (d=3,β=0.2). When D=1 and (d=3,β=0.2), the success threshold is 0.1758. It can be observed that the case of k/n=0.1 (below the success threshold) tends to 0 for smaller values of *l* compared with k/n=0.17 (near the success threshold), while the in the case of k/n=0.2 (above the success threshold) no more tend to 0. The simulation results of D=2 are also very similar to D=1, as shown in Figure 3. We observe that the two sets of results are in close agreement, particularly with the asymptotic analysis.

Notably, the above analysis is based on the assumption of a tree-like neighborhood. This is true when *n*, *k* goes to infinite. The concentration results and convergence to a tree-like case have been investigated widely in channel coding and CS [26,30], and our results in this regard are also consistent with [30], so, this content is omitted due to the limited space.

### 5.2. Finite Length Analysis of Integer Sum-Peeling Algorithm

This section evaluates the performance of an ISP algorithm with finite *n*. This is followed by a stopping set analysis of the decoding algorithm in binary erasure channel (BEC), but with some differences. In each iteration, the ISP algorithm can verify up to *D* nonzero variable nodes from a check node, while the traditional peeling algorithm can only decode, at most, one erasure in BEC. In order to exploit the similarity between the ISP algorithm and the traditional algorithm used on BEC, we expand Definition 4 to a generalized stopping sets.

**Definition** **5** **(*D*-stopping set).** *A D-stopping set $S_{D}$ is a subset of the set of variable nodes V such that all neighbors of $S_{D}$ are connected to $S_{D}$ at least D+1 times.*

The next lemma shows the key role that *D*-stopping set play in the ISP algorithm.

**Lemma** **2.** *Let regular (λ,ρ)-H constructed in (5) be a measurement matrix. Let T denote the set of nonzero coefficients of sparse binary signal. Then the set of nonzero variable nodes which remains when the sum-peeling algorithm stops is equal to the unique maximal D-stopping set of T.*

**Proof.**  Let $S_{D}$ be a *D*-stopping set included in T. Consider a special case when all other coefficients are known, every neighbor of $S_{D}$ has at least D+1 connections to the set $S_{D}$ so that the ISP algorithm can not verify the nonzero variable nodes included in the unique maximal stopping set contained in T. Conversely, if the ISP algorithm stops at a set $S_{D}$, then this happens only if all neighbors of $S_{D}$ have at least D+1 connections to $S_{D}$. Additionally, since zero variable nodes included in a *D*-stopping set can be verified by the ISP algorithm, $S_{D}$ must be maximal *D*-stopping set. □

Lemma 2 gives a foundation to implement the theoretical analysis and exploit the similar ensemble of LDPC results in BEC [31]. In [31], the authors developed combinatorial approaches to compute recursively the exact average bit erasure probability performance of the LDPC codes. Following these combinatorial approaches, the average unverified variable node probability (equivalent to bit erasure probability in BEC) can be calculated exactly. In the simulation results, we show the average verified nonzero variable node probability as a function of density factor α for finite *n*.

### 5.3. The Number of Measurements and Computational Complexity of Integer Sum Peeling Algorithm

**The number of measurements:** The ISP architecture (see Definition 2 (measurement matrix) in Section 3) with *d* stages, has *d* distinct measurement patterns, chosen as per the discussions in Section 3 and Section 4. These constructions are deterministic and are pre-computed. We assume that there is random access memory, and the unit cost of each I/O operation is used to read measurements. As shown in Section 4.1, we have three different ISP architectures, with d=3, d=4 and d=6 regions, for different *D*. The total measurements used for these regions are m=1.182k, m=1.2626k, and m=1.5456k, respectively, for D=1 and β=0.1. In general, for any fixed *d* and *D*, the number of measurements *m* can be as small as O(k).

It can be easily concluded that the proposed ISP requires less measurements than the existing method [14,19], which the number of required measurements is of O(−nlog(1−k/n)) and O(klog(n)). We also verify the measurement dimension required by these methods in simulations.

**The computational complexity:** In the proposed recovery algorithm, the maximum value of *D* is only considered to be 2. As shown in [24], the arithmetic operation is of O(k) for traditional CS. When *D* is set to 1, the main difference between ISP and traditional CS [24] is the rule **R2**, where the ISP algorithm requires extra q−1 comparisons. Thus, the recovery complexity is of O(k(q−1)).

It also can be easily concluded that the proposed ISP requires less iterations than the existing method [14,19], which the number of required iterations is of O(−nlog(1−k/n)) and O(klog(n)).

## 6. Noisy Measurements

We consider the following model of measurements contaminated with noise:

(12) y=Hx+n,

where the measurements y of (1) were corrupted by an additive noise vector n. The noise vector to the measurements results in the probability of having a 0 or integer amplitude of measurement to be zero. This will disable the rules **R1**, **R2** and **R3** in verifying the variable nodes. However, the rules can be effective by slight refinements.

We firstly show how to generalize the measurement matrix *H* of (5) in the noisy case. Following with (5), the m×n measurement matrix is given by

(13) $$H=\left[\begin{array}{c} {\tilde{h}}_{1}⊗F\\ \vdots \\ {\tilde{h}}_{m^{\prime }}⊗F \end{array}\right],$$

where the T×n matrix *F* is

$$F=\left[\begin{array}{cccc} 1 & \cdots & 1 & \cdots \\ 1 & \cdots & W^{i-1} & \cdots \\ \vdots & \vdots & \vdots & \vdots \\ 1 & \cdots & W^{\left(i-1)(T-1\right)} & \cdots \end{array}\right]\left[\begin{array}{cccc} g_{1} & 0 & \cdots & 0\\ 0 & g_{2} & \cdots & 0\\ \vdots & \vdots & \ddots & \vdots \\ 0 & \cdots & 0 & g_{n} \end{array}\right],$$

$m=Tm^{\prime }$, $W=e^{-j2\pi /p}$ (p⩾n is a prime), ${\tilde{h}}_{i}$ be the *i*-th row of the $m^{\prime }\times n$ local matrix $\tilde{H}$ and the weight entry $g_{i}$ is from i.i.d Gaussian distribution. The main difference between the construction of (5) and (13) is single vector f to matrix *F*, this results in single measurement $y_{i}$ to *T* measurements $y_{i},\cdots ,y_{i+T-1}$ (denotes as $y_{T}$ vector). Next, we exploit a similar rule of a noiseless case based on $y_{T}$.

**R4**. If ${∥y_{T}-F_{\left\{N(i)\right\}}\Lambda_{l}∥}^{2}⩽\left(1+\varepsilon \right)\sigma^{2}$, where $F_{\left\{N(i)\right\}}$ is taken from the columns of matrix *F* with support set N(i), $\Lambda_{l}$ is a binary vector of dimension $\left|N(i)\right|$ with at most *D* ones, $\sigma^{2}$ is variance of noise vector and ε is a parameter between 0 and 1. Then, all the variable nodes connected to $y_{T}$ are verified as $\Lambda_{l}$.

Similarly, the rule **R4** is responsible for verifying the variable nodes via the peeling–decoding algorithm. Therefore, the reconstruction algorithm in a noisy case is essentially identical with the noiseless case, i.e., the ISP algorithm. We will show the validity of the ISP algorithm with **R4** in the next section.

## 7. Simulation Results

In this section, we evaluate the performance of ISP and conventional sparse event detection algorithms through numerical simulations. We also show the degree of agreement between the asymptotic analysis and simulation results. In all simulations, the left *d*-regular bipartite graph G(d,β) (β=m/n) is constructed randomly.

Firstly, we compare the proposed ISP algorithm with conventional basis pursuit (BP) algorithm in CS [1] and the sum of absolution values (SAV) algorithm [17]. Three event sources cases of X are considered (same with [17]): $X_{1}=\left\{0,1\right\},P\left(0\right)=\mathrm{Pr},P\left(1\right)=1-\mathrm{Pr}$; $X_{2}=\left\{-1,0,1\right\},P\left(0\right)=\mathrm{Pr},P\left(1\right)=P\left(-1\right)=\left(1-\mathrm{Pr}\right)/2$; $X_{3}=\left\{-2,-1,0,1,2\right\},P\left(0\right)=\mathrm{Pr},P\left(-2\right)=P\left(-1\right)=P\left(1\right)=P\left(2\right)=\left(1-\mathrm{Pr}\right)/4$. In [17], the author considers the finite alphabet set X that does not necessarily contain 0, i.e., 0⩽Pr⩽1. In this paper, we only consider the sparse integer-valued signal with high Pr. In the SAV and BP algorithms, the simulations are based on randomly generated measurement matrix $H\in R^{200\times 1000}$ whose entries are chosen independently from standard Gaussian distribution. In the proposed ISP algorithm, the measurement matrix $H\in C^{100\times 1000}$ is adopted as constructed in (5) with the bipartite graph G(3,0.1).

**Figure 4:**
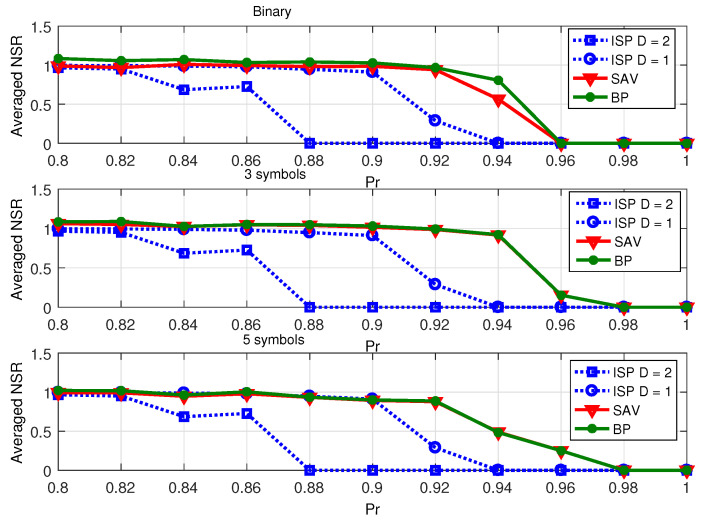
Averaged NSR performance of sparse recovery algorithms for integer-valued signal.

Figure 4 illustrates the averaged NSR (noise-to-signal ratio) ${∥x-\hat{x}∥}_{2}/{∥x∥}_{2}$ as a function of the probability Pr of zero elements in X. Note that the lower critical probability Pr implies a better recovery performance. As shown in the figure, the critical probability Pr of the proposed ISP algorithm (D=1 and D=2) is lower than the BP and SAV algorithms for all cases of X. Furthermore, the performance of the proposed ISP algorithm is independent of the number of symbols *q*, while the BP and SAV algorithms have worse performance, with decreased *q*.

**Figure 5:**
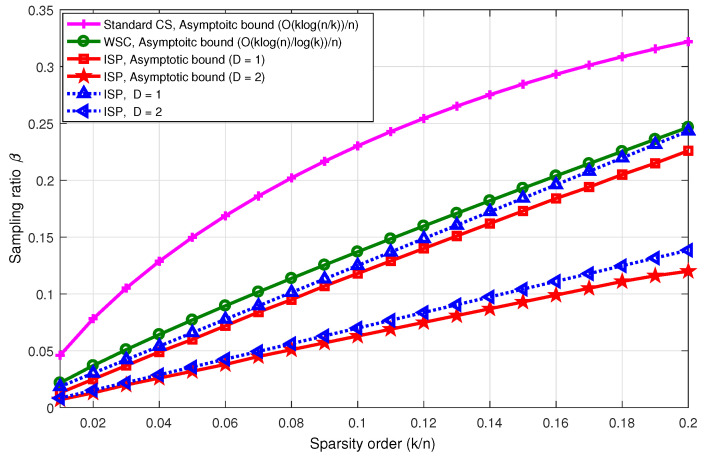
The minimum sampling ratio required to exactly recover the integer-valued sparse signal of dimension $n=10^{4}$ versus the sparsity order.

Secondly, we show the number of measurements (the number of sensors) required to exactly detect the sparse events versus the sparsity order. Each nonzero element of x is randomly chosen from the event sources set $X^{*}=\left\{+1,-1\right\}$. The support of x (the position of active events) is chosen randomly, d=3 is adopted, the $n=10^{4}$ event sources are randomly distributed in an area of 1000 m by 1000 m, and the propagation loss factor is 3. As shown in Figure 5, the number of sensors of the proposed ISP method is significantly lower than that for the asymptotic bound of the WSC method [7] and standard CS (D=1 and D=2). In addition, as see in the figure, the simulation results are close with the asymptotic threshold.

Thirdly, we apply the ISP algorithm to six randomly constructed left *d*-regular bipartite graphs G(d=6,β=0.05) with event sources $n=3\times 10^{3},\;10^{4},\;10^{5}$ to investigate the agreement between our asymptotic analysis and simulation results. Each nonzero element of x is randomly chosen from the set $X^{*}=\left\{+1,-1\right\}$. Let $p_{0}$ denote the maximum sparsity order for the signal of length *n*, to be exactly recovered by the proposed ISP algorithm. The exact recovery of thresholds $p_{0}$ are listed in Table 2. As can be seen, for all graphs, the larger *n* indicates higher closeness between the thresholds $p_{0}$ and the asymptotic threshold. Accordingly, the simulation results match well with the theoretical results.

Fourthly, we compare the performance of the ISP algorithm with that of existing binary event detection algorithms considering binary event detection, a special class of event sources.

**Figure 6:**
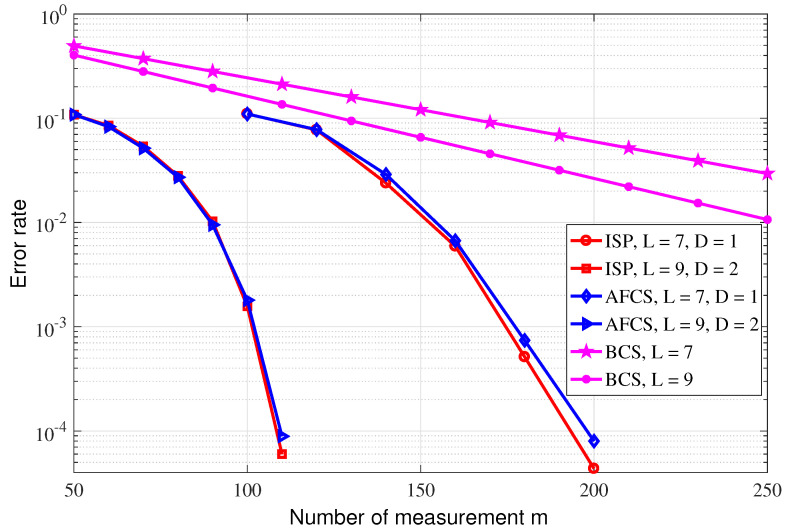
Performance comparison between ISP and other existing binary signal reconstruction algorithms.

**Figure 7:**
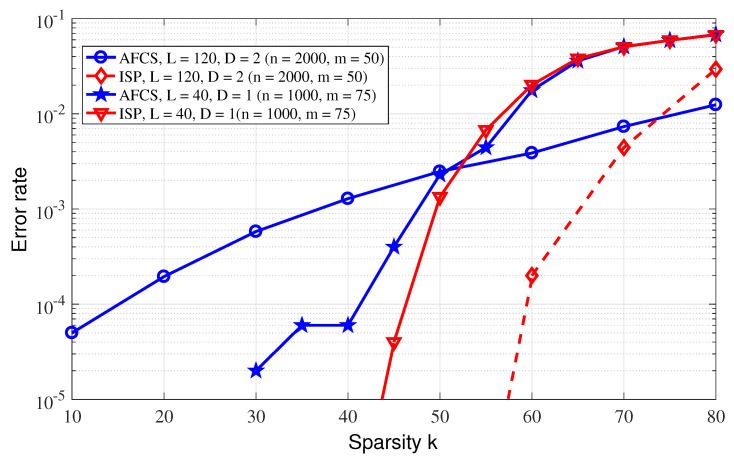
Performance comparison between ISP and AFCS reconstruction algorithm with fixed row weight.

**Figure 8:**
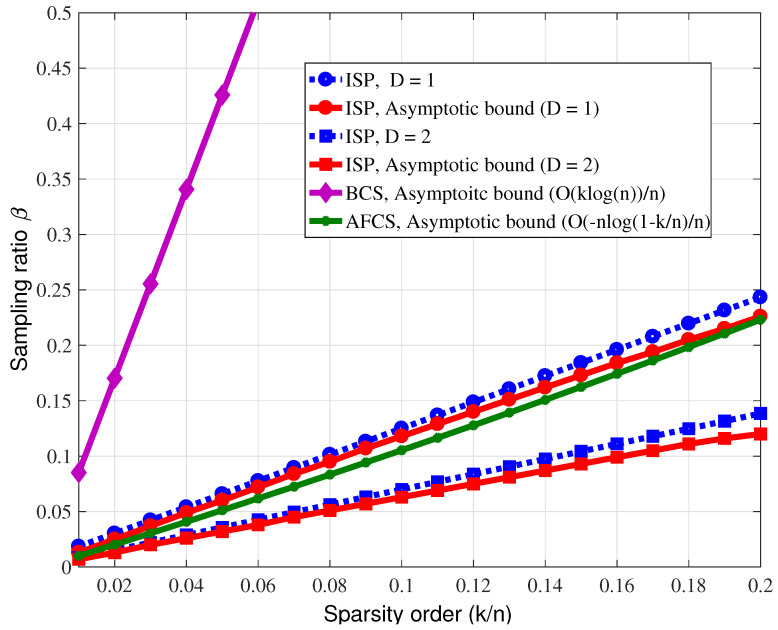
Minimum sampling rate required for accurate reconstruction of binary sparse signals.

(1) Consider that the n=500 event sources are randomly distributed in an area of 500 m by 500 m, the propagation loss factor is 3 and the sparsity *k* is set to 100. The optimal row weights of the sparse matrix corresponding to AFCS are set, respectively, when D=1, L=7, and when D=2, L=9. ISP and BCS also employ the sparse matrices of the AFCS scheme for a fair comparison. Figure 6 shows the reconstruction error rate performance curves of the ISP algorithm and the two existing reconstruction algorithms of AFCS and BCS with the measurement dimension varying. The reconstruction error rate is defined as the ratio of the number of nonzero signals that were not successfully reconstructed to the signal dimension. From Figure 6, it can be observed that the performance of the proposed ISP algorithm is basically the same as that of the AFCS algorithm; however, previous analysis reveals the lower complexity of the ISP algorithm compared with the AFCS algorithm. On the other hand, it can also be observed that both the ISP and AFCS reconstruction algorithms outperform the BCS scheme.
(2) In the AFCS algorithm, the optimal row weight of the sparse matrix is $L=\left\lceil -0.5(D+2)/log(1-k/n)\right\rceil$; however, the AFCS scheme generally cannot optimize the row weight of its measurement matrix because of the actual unknown or variable sparsity. In this experiment, we fix the row weight of the sparse matrix, and compare the performance of the ISP and AFCS algorithms. Consider two experimental situations, L=40, event sources n=1000 and L=120, event sources n=2000. Figure 7 reveals the better performance of the ISP algorithm compared with the AFCS algorithm, for a fixed row weight, which suggests that the ISP scheme is robust to sparsity varying. In addition, it can also be observed that there is a broken line in AFCS. This is because the AFCS constructs the measurement matrix adaptively, and when the row weight is not optimal, there may be no solution.
(3) The theoretical performance of the above three event detection algorithms is also compared. In the simulation, event sources $n=10^{5}$ are randomly distributed in an area of 1000 m by 1000 m, the propagation loss factor is 3. The measurement matrix is a randomly generated left-regular sparse matrix, where the column weight is d=3. According to [14], it can be observed that the measurement dimension required by AFCS to accurately reconstruct sparse binary signals is O(−nlog(1−k/n)), which is smaller than O(klog(n)) that required by BCS. Figure 8 demonstrates the lower sampling rate required for accurate reconstruction of the ISP algorithm proposed in this paper compared with the BCS and AFCS schemes when D=2, and the performance of the ISP algorithm and AFCS algorithm are basically identical when D=1. Furthermore, it can be found that the simulation results of the ISP reconstruction algorithm are basically consistent with the theoretical results.

Finally, we show the validity and performance of the ISP algorithm in a noisy environment. The binary sparse event sources are adopted for ease of comparison. The signal-to-noise ratio (SNR) is defined as:

(14) $$\mathrm{SNR}=10\log_{10}\frac{{∥\mathrm{Hx}∥}^{2}}{{∥n∥}^{2}}\;\mathrm{dB},$$

**Figure 9:**
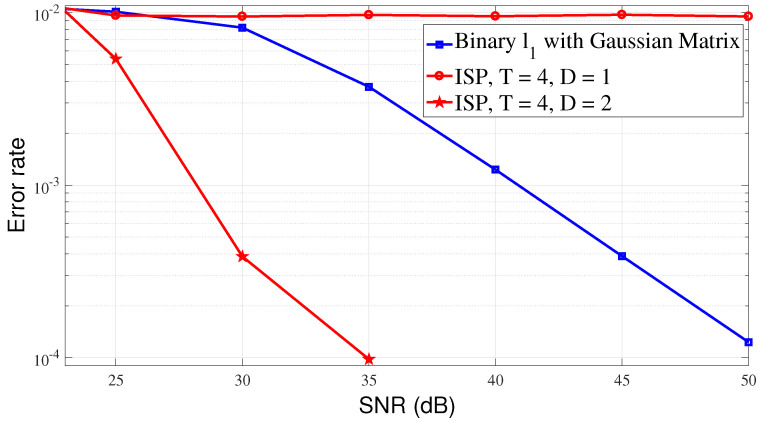
MSE performance under ISP and $\ell_{1}$ reconstruction algorithm.

Figure 9 illustrates the MSE performance of the ISP and binary $\ell_{1}$ reconstruction algorithms as a function of SNR. For the binary $\ell_{1}$ reconstruction algorithm, a standard Gaussian random matrix is taken, and for the ISP algorithm, the rows of the sparse matrix are reset to 3, T=4. In all simulation experiments, event sources $n=10^{3}$ are randomly distributed in an area of 500 m by 500 m, the propagation loss factor is 3, the matrix measurement dimension is 100×1000, and the sparsity is set to k=20. According to the figure, it can be observed that the MSE performance of the ISP algorithm is better than that of the event detection algorithm when D=2. This also means that the ISP algorithm is also effective in noisy environments.

## 8. Conclusions

In this paper, the event-detection problem is modeled as the decoding of sparse graph code, and the problem in the IoT system is described from the perspective of coding theory. Using compressive sensing, the high-dimensional event sources are recovered from incomplete linear measurements. A simple deterministic sparse measurement matrix was adopted to uniquely determine the event sources, based on which an integer sum-peeling recovery algorithm was proposed to exploit the structure of event detection. To corroborate the performance of the recovery algorithm, the asymptotic analysis was provided via the density evolution, which was verified by simulation. Simulation results also showed that the event detection algorithm had superior performance compared with other existing algorithms.

## Figures and Tables

**Table 1:** Success thresholds of ISP for different (d,β).

(d,β)	(3, 0.1)	(3, 0.2)	(4, 0.1)	(4, 0.2)	(6, 0.1)	(6, 0.2)
D=1	0.0846	0.1758	0.0792	0.1629	0.0647	0.1319
D=2	0.1627	0.3444	0.1378	0.2867	0.1043	0.2136

**Table 2:** Asymptotic and simulation success thresholds for ISP.

*D*	$3\times 10^{3}$	$1\times 10^{4}$	$1\times 10^{5}$	Asymptotic
1	$p_{0}=0.0013$	$p_{0}=0.0023$	$p_{0}=0.003$	$p_{0}=0.0031$
2	$p_{0}=0.0027$	$p_{0}=0.0038$	$p_{0}=0.0047$	$p_{0}=0.0051$

## Data Availability

Not applicable.
